# Clinical subtype of HAM/TSP based on clinical course and laboratory findings

**DOI:** 10.1186/1742-4690-8-S1-A42

**Published:** 2011-06-06

**Authors:** Yoshihisa Yamano, Tomoo Sato, Natsumi Araya, Naoko Yagishita, Yukiko Shimizu, Hitoshi Ando, Atae Utsunomiya, Shuji Izumo, Steven Jacobson, Noboru Suzuki

**Affiliations:** 1 Institute of Medical Science, St. Marianna University School of Medicine, Kawasaki, Japan; 2 Department of Hematology, Imamura Bun-in Hospital, Kagoshima, Japan; 3 Molecular Pathology, Center for Chronic Viral Diseases, Graduate School of Medical and Dental Sciences, Kagoshima University, Kagoshima, Japan; 4 Viral Immunology Section, Neuroimmunology Branch, National Institute of Health, Bethesda, MD, USA

## 

The clinical course and disease activity of patients with HTLV-1 associated myelopathy / tropical spastic paraparesis (HAM/TSP) are different among patients. Therefore, the treatment plan should be designed based on these backgrounds of patients. However, there is little information about the natural history of HAM/TSP and biomarkers of disease activity that is associated with prognosis.

As the candidate for biomarkers to evaluate the disease activity in HAM/TSP, HTLV-1 proviral load in PBMC, several cytokines and chemokines in serum or cerebrospinal fluid (CSF) are known to be increased in HAM/TSP patients. However, little is known which parameter of these candidates is most associated with disease severity.

Therefore, we investigated the clinical course of 30 HAM/TSP patients without any history of treatment. Furthermore, we measured quantitatively the concentration of a series of cytokines and chemokines in serum and CSF, and HTLV-1 proviral DNA load in PBMC. Then, the level of these markers was evaluated for the correlation with disease severity.

In HAM/TSP patients, the level of CXCL10/IP-10 and neopterin in CSF was strongly correlated with disease severity. Interestingly, the level of soluble IL-2 receptor and CXCL10/IP-10 in serum was also correlated with disease severity with statistical significance. Furthermore, based on the clinical course and laboratory findings, HAM/TSP was classified into 4 different clinical subtypes as follows; (1) Rapidly progressive (active), (2-A) Chronic progressive (active), (2-B) Chronic progressive (inactive), (3) Chronic mild (inactive). This classification might be useful to determine the therapeutic strategy for patients with HAM/TSP.

